# Squamous cell carcinoma antigen (SCCA) is up-regulated during Barrett’s carcinogenesis and predicts esophageal adenocarcinoma resistance to neoadjuvant chemotherapy

**DOI:** 10.18632/oncotarget.14108

**Published:** 2016-12-22

**Authors:** Matteo Fassan, Stefano Realdon, Luca Vianello, Santina Quarta, Alberto Ruol, Carlo Castoro, Marco Scarpa, Giovanni Zaninotto, Vincenza Guzzardo, Vanna Chiarion Sileni, Patrizia Pontisso, Massimo Rugge

**Affiliations:** ^1^ Department of Medicine (DIMED), Surgical Pathology Unit, University of Padua, Padua, Italy; ^2^ Gastroenterology Unit, Istituto Oncologico Veneto, IOV-IRCCS, Padua, Italy; ^3^ Department of Medicine (DIMED), 5th Medical Clinic, University of Padua, Padua, Italy; ^4^ Department of Surgical, Oncological and Gastroenterological Sciences (DiSCOG), 3rd Surgical Clinic, University of Padua, Padua, Italy; ^5^ Esophageal and Digestive Tract Surgical Unit, Veneto Institute of Oncology, IOV-IRCCS, Padua, Italy; ^6^ Imperial College London, Department of Surgery and Cancer, Division of Surgery, London, UK; ^7^ Melanoma & Esophageal Oncology Unit, Istituto Oncologico Veneto, IOV-IRCCS, Padua, Italy; ^8^ Veneto Tumour Registry, Veneto Region, Padua, Italy

**Keywords:** SCCA, esophageal adenocarcinoma, Barrett's carcinogenesis, preneoplastic lesions

## Abstract

Squamous Cell Carcinoma Antigen (SCCA) is consistently overexpressed in many different solid tumors, and has been associated with both tumor aggressiveness and chemoresistance. No data, however, is currently available on SCCA expression during esophageal Barrett's carcinogenesis, nor on SCCA expression's role on esophageal adenocarcinoma chemoresistance. The SCCA immunohistochemical expression was assessed in a series of 100 biopsy samples covering the whole histological spectrum of Barrett's oncogenesis. Squamous native mucosa was characterized by a moderate to strong cytoplasmic and nuclear SCCA expression in suprabasal, medium, and superficial layers. On the other hand, almost half of the considered lesions did not express SCCA; the other half featured weak to moderate SCCA expression. The relationship between SCCA protein expression and tumor response to neoadjuvant chemotherapy was assessed in 90 esophageal adenocarcinoma specimens (40 biopsy and 50 surgery specimens), stratified according to Mandard tumor regression grade. As observed in other settings, the presence of SCCA expression clustered in the group of tumors characterized by a lower responsiveness to neoadjuvant treatments. The present results suggest an involvement of SCCA in a subset of Barrett-related tumors, and prompt to consider the SCCA-protein expression as response-predictive marker of neoadjuvant therapy in esophageal adenocarcinomas.

## INTRODUCTION

Barrett's esophagus is a complication of longstanding gastro-esophageal reflux and is a major risk for esophageal adenocarcinoma, one of the leading causes of cancer-related death worldwide [1–4].

The recent advances in the therapeutic approaches for gastroesophageal tumors has significantly improved the curative resection rates, and both the disease-free and the overall survival [5–7]. Nevertheless, no biomarker is available to reliably predict tumor chemosensitivity.

Squamous cell carcinoma antigen (SCCA), including its two isoforms SCCA-1 (also known as SerpinB3) and SCCA-2 (also known as SerpinB4), belongs to the clade B subset of serpins [8–10]. The first described isoform, SCCA-1, was initially found to be significantly overexpressed in carcinomas with squamous differentiation and in hepatocellular carcinoma [11–13]. Subsequently, other studies pinpointed a significant association between SCCA-1 overexpression, more aggressive tumor phenotypes and chemoresistance in different tumor types [8, 14–16].

SCCA-1 confers to cancer cells resistance to induced apoptosis by different mechanisms, including inhibition of lysosomal cathepsin proteases, JNK pathway or p38 activation [8]. More recently, it has been demonstrated the role of SCCA-1 in inhibiting reactive oxygen species (ROS) generation and cell death through its inhibitory interaction with respiratory Complex I [14].

SCCA expression has never been investigated in the different histology phenotypes of the multistep Barrett's carcinogenetic process. We first analyzed SCCA expression by immunohistochemistry on a series of Barrett-related lesions. We also focused on the relationship between SCCA expression and tumor response to neoadjuvant chemotherapy, as assessed by Mandard scale Tumor Regression Grade (TRG) [17].

## RESULTS

### SCCA expression is downregulated in Barrett's related lesions in comparison to squamous esophageal mucosa

SCCA-1 and SCCA-2 expression (mRNA) were assessed in native squamous esophageal mucosa (N), Barrett's mucosa (BM), and Barrett's adenocarcinoma (BAc) by investigating the results of four Barrett's adenocarcinoma microarray data sets through the Oncomine and the NCBI-GEO websites [18–23].

**Figure 1:**
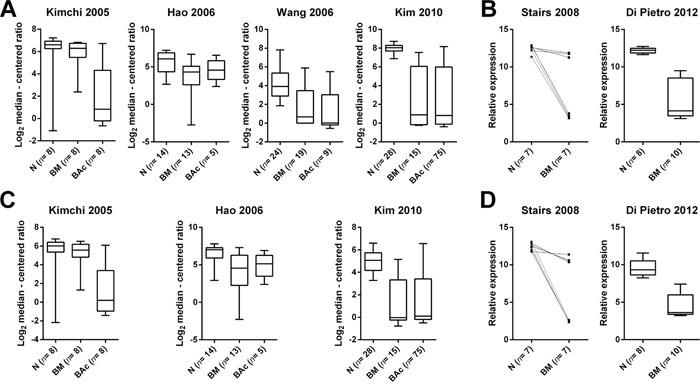
SCCA expression is downregulated in Barrett's related lesions in comparison to squamous esophageal mucosa, but SCCA expression prevalence is consistent among the different lesion types **A**. SCCA-1 expression (mRNA) in normal squamous esophageal mucosa (N), intestinalized Barrett's mucosa (BM), and Barrett's adenocarcinoma (BAc) as obtained by analyzing the results of four Barrett's adenocarcinoma microarray data sets [20–23]. Statistical significance was calculated using the Oncomine website (www.oncomine.org). SCCA-1 expression was significantly downregulated in BM and BAc in comparison to N in 3 out of 4 studies (Kimchi: N *vs* BM *p*=ns, N *vs* BAc *p*=0.012; Hao: N *vs* BM *p*=0.013, N *vs* BAc *p*=ns; Wang: N *vs* BM *p*<0.001, N *vs* BAc *p*<0.001; Kim: N *vs* BM *p*<0.001, N *vs* BAc *p*<0.001). Boxplots represents distribution of normalized data obtained from the Oncomine database. **B**. Two independent studies in the NCBI GEO database (http://www.ncbi.nlm.nih.gov/geo/) showed a significant down-regulation of SCCA-1 expression in BM *versus* N (*p*=0.016 and *p*<0.001, respectively) [18, 19] (relative expression= distribution of normalized data obtained from the GEO database). **C**. SCCA-2 expression was significantly downregulated in BM and BAc in comparison to N in all the three studies analyzed (Wang study had no data for SCCA-2; Kimchi: N *vs* BM *p*=ns, N *vs* BAc *p*=0.004; Hao: N *vs* BM *p*=0.003, N *vs* BAc *p*=0.040; Kim: N *vs* BM *p*<0.001, N *vs* BAc *p*<0.001). **D**. A significant downregulation of SCCA-2 in BM *versus* N was observed in both studies available at the NCBI GEO database (*p*=0.010 and *p*<0.001, respectively).

SCCA-1 expression was significantly downregulated in BM and BAc in comparison to N in 3 out of 4 Oncomine studies (Kimchi: N *vs* BM *p*=ns, N *vs* BAc *p*=0.012; Hao: N *vs* BM *p*=0.013, N *vs* BAc *p*=ns; Wang: N *vs* BM *p*<0.001, N *vs* BAc *p*<0.001; Kim: N *vs* BM *p*<0.001, N *vs* BAc *p*<0.001) [20–23] (Figure 1A). The fourth non-significant comparison showed, however, a trend in SCCA-1 mRNA down-regulation in lesion samples compared to squamous esophageal mucosa. Non-esophageal samples, considered as normal samples in the original studies (*i.e*., small intestine samples), were not retained in the analysis.

By exploring the NCBI-GEO database, we analyzed two further independent studies, which both showed a significant down-regulation of SCCA-1 expression in BM *versus* N (*p*=0.016 for matched samples and *p*<0.001, respectively) [18, 19] (Figure 1B).

SCCA-2 was similarly deregulated in both BM and BAc. In particular, SCCA-2 expression was significantly downregulated in BM and BAc samples in comparison to native squamous mucosa in all the three available Oncomine studies analyzed (Kimchi: N *vs* BM *p*=ns, N *vs* BAc *p*=0.004; Hao: N *vs* BM *p*=0.003, N *vs* BAc *p*=0.040; Kim: N *vs* BM *p*<0.001, N *vs* BAc *p*<0.001) samples (Figure 1C). A similar significant downregulation of SCCA-2 in BM *versus* N was observed in both studies available at the NCBI GEO database and previously characterized for SCCA-1 expression (*p*=0.010 and *p*<0.001, respectively) (Figure 1D).

### SCCA expression prevalence is consistent among the different Barrett’s-related lesion subtypes

**Figure 2:**
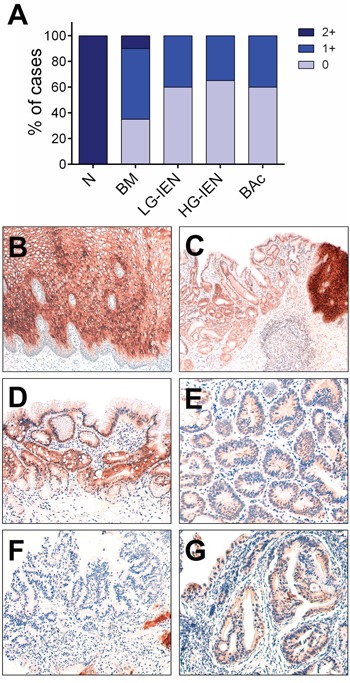
SCCA immunohistochemical expression during Barrett's carcinogenesis **A**. SCCA immunohistochemical expression distribution during Barrett's carcinogenesis; 20 cases per group were considered. LG-IEN= low-grade intraepithelial neoplasia; HG-IEN= high-grade intraepithelial neoplasia. **B**. Representative IHC stains showing a moderate to strong cytoplasmic and nuclear SCCA expression in suprabasal, medium, and superficial layers of normal esophageal squamous mucosa. Note that basal layers were SCCA negative. **C**. SCCA expression at squamous-columnar junction; on the right the positive esophageal mucosa, on the left the mild to moderate stained Barrett's mucosa. **D**. Intestinalized Barrett's mucosa epithelia showing a moderate SCCA expression. **E**. A SCCA faintly stained low-grade intraepithelial lesion. **F**. A negative high-grade intraepithelial neoplasia with some SCCA positive esophageal mucosa remnants. **G**. A faint SCCA positive early Barrett's adenocarcinoma. (Original magnifications 4x, 10x, and 20x).

SCCA expression was analyzed by immunohistochemistry on a series of 100 different endoscopy biopsy samples representative of normal esophageal mucosa and each of the phenotypic lesions occurring in the Barrett's oncogenic cascade (N= 20 cases; BM= 20 cases; low-grade intraepithelial neoplasia [LG-IEN]= 20 cases; high-grade intraepithelial neoplasia [HG-IEN]= 20 cases; BAc= 20 cases) (Figure 2A).

Normal esophageal mucosa showed moderate to strong cytoplasmic and nuclear SCCA expression in suprabasal, medium, and superficial layers (*i.e*., spinous and granular layers), whereas basal layers were SCCA negative (Figure 2). As observed in the array database meta-analysis for both SCCA isoforms, SCCA expression was significantly down-regulated in the Barrett-related lesions (*p*=ns). However, a comparable SCCA expression prevalence was observed among groups, with around 40-60% of samples showing a 1+ SCCA expression. Most of these samples expressing SCCA showed a faint to moderate cytoplasmic immunoreaction. Two BM samples showed a strong 2+ cytoplasmic immunoreaction. No significant relationship was observed among SCCA expression and the intensity of the inflammatory infiltrate in BM and neoplastic lesions.

### SCCA-expressing tumors are less sensitive to neoadjuvant chemotherapy

**Figure 3:**
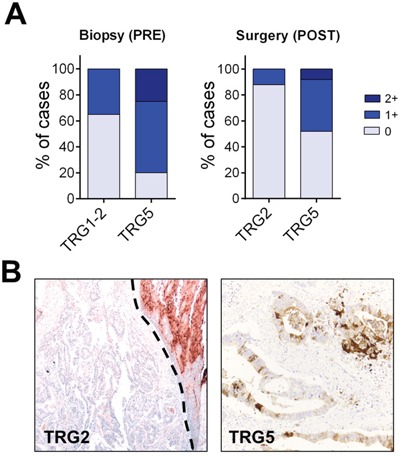
Tumors expressing SCCA are less sensitive to neoadjuvant chemotherapy **A**. SCCA immunohistochemical distribution in pre-neoadjuvant therapy endoscopy biopsy and post-therapy surgical samples. SCCA-expressing tumors as both assessed in endoscopy biopsies or surgical specimens was associated to a lower sensitivity to neoadjuvant treatments (both *p*<0.001). **B**. Representative IHC examples on endoscopy biopsy specimens of a SCCA-negative TRG2 tumor (upper right the normal squamous esophageal mucosa) and a SCCA 2+ TRG5 adenocarcinoma. Note the heterogeneous SCCA expression pattern observed in this tumor. (Original magnifications, 4x and 20x).

Since SCCA-1 overexpression has been associated to a worst prognosis and chemoresistance in several different tumor types [8, 14–16, 24], we assessed SCCA expression in a series of BAc specimens selected according to their post-neoadjuvant chemotherapy Mandard status (Figure 3A).

The presence of SCCA expression (both 1+ and 2+) as both assessed in pre-neoadjuvant therapy endoscopy biopsies (*n*=40; 20 TRG1/2 and 20 TRG5) or post-therapy surgical specimens (*n*=50; 25 TRG2 and 25 TRG5) was significantly associated to a lower sensitivity to neoadjuvant treatments (both *p*<0.001). Of note, all the observed 2+ cases clustered in the Tumor Regression Grade 5 (TRG5) group. Two out of five 2+ SCCA cases showed a strong cytoplasmic (and focally nuclear) heterogeneous reaction (Figure 3B).

## DISCUSSION

In Western countries, the rising incidence of esophageal adenocarcinoma is associated with an overall 5-year survival rate lower than 20% [1, 25]. Currently, neoadjuvant chemo(radio)therapy followed by surgical resection offers the best clinical outcome for locally advanced tumors, with around 25% of complete response rate [5, 26–28]. Moreover, the pathologic complete response is associated with lower recurrence, lower metastatic potential, and longer overall survival [29]. No reliable prognostic biomarker, however, is currently available to identify those tumors that may benefit of neoadjuvant therapeutic protocols.

This study focuses on SCCA expression in Barrett's carcinogenesis. Under normal conditions, SCCA expression is reliably expressed in both the spinous and the granular layers of normal squamous epithelium (tongue, esophagus, tonsil, cervix uterine, vagina, Hassal's corpuscles of the thymus and some areas of the skin) [30, 31]. As expected, also in the present study, native esophageal mucosa consistently featured SCCA expression.

In both microarray data metanalysis and immunohistochemistry, we observed a significant reduction in SCCA expression in Barrett's related lesions in comparison to normal mucosa. Of note, however, a significant proportion of the considered cases consistently retained a weak to moderate protein expression, with no significant phenotypic differences among SCCA-positive and SCCA-negative cases.

A similar SCCA downregulation has been demonstrated in the esophageal squamous cell carcinoma (ESCC) setting in both invasive and intraepithelial lesions by proteomic analysis [32]. These data further emphasize the importance of SCCA expression maintenance in a subset of esophageal tumors. On the other hand, a significant overexpression in circulating SCCA-2 mRNA levels were observed among ESCC patients [33], but the cell source (i.e., SCCA-expressing tumors and/or normal esophageal mucosa damaged by neoplastic cells) should be further explored.

Increased SCCA-1 expression has been recently associated to inhibition of protein turnover, unfolded protein response, and activation of NF-kB signaling [34]. Of note, NF-κB pathway has been associated with BE-related cancer progression, being up-regulated in BM and BAc samples [35]. In this setting, it regulates the expression of many pro-inflammatory and growth regulatory cytokines including tumour necrosis factor-α(TNF-α). Confirming these data, TNF-α expression has been shown to increase along the metaplasia-dysplasia-carcinoma sequence, leading to an increase in the proto-oncogene MYC via a β-catenin mediated pathway [36]. A SCCA-1-mediated (both direct and indirect) MYC overexpression has recently been demonstrated in hepatocellular carcinoma [37]. Overall, these data suggest a possible role for SCCA-1 in the neoplastic transformation of a distinct sub-group of Barrett's mucosa.

SCCA-1 is highly expressed in the spinous and granular layers of normal skin, which is histologically similar to what observed in the esophageal mucosa. Katagiri and colleagues recently described that SCCA-1 levels are significantly up-regulated in lesional skin compared to their normal sun-protected skin [38]. Furthermore, subjects with high levels of SCCA-1 in the epidermis were more susceptible to barrier disruption by external stimuli, and this was accompanied with a further subsequent increase of SCCA-1. This model could be translated into esophageal pathophysiology: the gastro-esophageal reflux will lead to a chronic inflammatory insult to the esophageal mucosa with a concurrent SCCA-1 up-regulation and disruption of the esophageal mucosa barrier to external stimuli, with an accelerated process of mucosa metaplastic transformation. On the other hand, metaplastic epithelia are SCCA-1 negative. As observed in liver carcinogenesis, under longstanding inflammation the metaplastic Barrett-epithelia would acquire a progressive increase in SCCA-1 expression, which would sustain the oncogenic process within the Barrett's mucosa [39].

Recent evidence linked SCCA-1 overexpression to chemoresistance in several different tumor types [8, 14–16, 24]. Based on such features, in a series of BAc cases we explored the relationship between SCCA expression and the tumor response to neoadjuvant chemotherapy (as assessed by Mandard score). Of interest, most of tumors with a SCCA expression clustered in the TRG5 group, demonstrating a significant association between the SCCA expression and a lower responsiveness to neoadjuvant treatments. Similar data have been observed in epithelial ovarian cancers exposed to platinum-based chemotherapy regimens [16, 40] and in breast carcinomas treated with anthracycline-based neoadjuvant chemotherapy [15]. In addition, it has recently been demonstrated that SCCA-1 protects from oxidative damage by chemotherapeutics through inhibition of mitochondrial respiratory complex I [14].

In conclusion, this study originally demonstrated a significant downregulation of the SCCA expression in the different phenotypic lesions included in the Barrett's carcinogenesis. In esophageal adenocarcinoma, the significant association between SCCA-expression and a lower responsiveness to neoadjuvant chemotherapy, potentially qualifies the protein expression as a prognostic-predictive marker to select patients and define more effective and specific interventional approaches.

## MATERIALS AND METHODS

### Ethics statement

All the histopathological samples were retrospectively collected (2006-2015) from the files of the Surgical Pathology Unit of the Department of Medicine (DIMED) at the University of Padua and from the Veneto Region's multicenter Barrett Esophagus Registry (EBRA, Padua Unit) [41]. Investigation has been conducted in accordance with the ethical standards, according to national and international guidelines, and has been approved by the authors’ institutional review board (418/04/CE).

### Materials

A total of 190 formalin-fixed paraffin-embedded tissue samples were considered and included in this study: 140 endoscopic biopsy samples (100 representative of the Barrett's carcinogenetic cascade and 40 selected according to their Mandard TRG) derived from different biopsy sets and 50 BAc samples obtained from surgical specimens (M/F 111/89; median age 62, 44-78; all Caucasian).

Biopsy samples were representative of each of the phenotypic lesions characterizing Barrett's carcinogenic cascade [2, 3], and were obtained from histologically confirmed long-segment Barrett esophagus patients (*i.e*., Barrett's mucosa >3cm in length): (i) 20 biopsy samples of Barrett's mucosa with intestinal metaplasia (BM; ≥75% of the glands); (ii) 20 biopsy samples of Barrett low-grade intraepithelial neoplasia (LG-IEN); (iii) 20 biopsy samples of Barrett high-grade IEN (HG-IEN); (iv) 20 biopsy samples of well- to moderately differentiated BAc (all G1/G2 and pT1 or pT2 cancers). For control purposes, 20 further native esophageal mucosa samples (N), as obtained form 20 dyspeptic patients, were considered.

To test SCCA expression predictive impact on platinum-based neoadjuvant chemotherapy, a series of 90 esophageal adenocarcinoma specimens stratified according to Mandard tumor regression grade (TRG) was considered [17]. Because of the high intra-pathologists variability in assessing Mandard score [38], only tumors characterized by TRG1/2 (*i.e*., TRG1= tumor complete regression; TRG2= fibrosis with scattered tumor cells) and TRG5 (*i.e*., TRG5= tumor without changes of regression) were selected. In particular two different series were analyzed: i) forty pre-therapy BAc biopsy samples selected according to the final TRG score on the surgically treated adenocarcinoma (20 TRG1/2 and 20 TRG 5); ii) fifty post-neadjuvant therapy surgically-treated adenocarcinomas (25 TRG2 and 25 TRG 5). In the latter group, only TRG2 cases were considered because TRG1 esophagectomies lack by definition tumor cells to be analyzed.

### Array database meta-analysis

The Oncomine database and gene microarray analysis tool, a repository for published microarray data (www.oncomine.org) [42], was explored (15 May 2016) for SCCA-1and SCCA-2 mRNA expression in esophageal native mucosa, BM and BAc samples. Oncomine algorithms, which enable multiple comparisons among different studies, were used for the statistical analysis of the differences in mRNA expression between the aforementioned comparisons.

The NCBI-GEO repository of published array data (http://www.ncbi.nlm.nih.gov/geo/) and the GEO2R microarray analysis tool were explored (15 May 2016) to assess SCCA-1and SCCA-2 expression in Barrett-related lesions (using the keywords: SCCA-1 or serpinB3, and esophagus). Studies already considered in the Oncomine analysis were excluded from the NCBI-GEO analysis.

### Immunohistochemistry (IHC)

The immunohistochemical expression of SCCA (polyclonal; rabbit; Hepa-Ab, Xeptagen, Venice, Italy) was performed on the automated Leica Microsystems Bondmax® (Leica, Wetzlar, Germany). This antibody recognizes both SCCA isoforms. Immunostaining was scored jointly by two pathologists (MF & MR). Both cytoplasmic and nuclear staining was retained for scoring. Immunostaining was semiquantified using a three-tier scoring based on intensity of staining (0= negative; 1= weak/moderate; 2= strong).

### Statistical analysis

Differences between groups were tested by applying the (paired) *t*-test, the modified Kruskal–Wallis nonparametric test for trend, and the Mann Whitney test, as appropriate. *P* values <0.05 were considered significant. The statistical analysis was performed using STATA software (Stata Corporation, College Station, TX).

## References

[1] Rustgi AK, El-Serag HB (2014). Esophageal carcinoma. N Engl J Med.

[2] Saraggi D, Fassan M, Bornschein J, Farinati F, Realdon S, Valeri N, Rugge M (2016). From Barrett metaplasia to esophageal adenocarcinoma: the molecular background. Histol Histopathol.

[3] Fassan M, Baffa R, Kiss A (2013). Advanced precancerous lesions within the GI tract: the molecular background. Best Pract Res Clin Gastroenterol.

[4] Spechler SJ, Souza RF (2014). Barrett’s esophagus. N Engl J Med.

[5] Cunningham D, Allum WH, Stenning SP, Thompson JN, Van de Velde CJ, Nicolson M, Scarffe JH, Lofts FJ, Falk SJ, Iveson TJ, Smith DB, Langley RE, Verma M, MAGIC Trial Participants (2006). Perioperative chemotherapy versus surgery alone for resectable gastroesophageal cancer. N Engl J Med.

[6] Fassan M, Mastracci L, Grillo F, Zagonel V, Bruno S, Battaglia G, Pitto F, Nitti D, Celiento T, Zaninotto G, Fiocca R, Rugge M (2012). Early HER2 dysregulation in gastric and oesophageal carcinogenesis. Histopathology.

[7] Fiocca R, Mastracci L, Milione M, Parente P, Savarino V, Gruppo Italiano Patologi Apparato Digerente (GIPAD), Società Italiana di Anatomia Patologica e Citopatologia Diagnostica/International Academy of Pathology, Italian division (SIAPEC/IAP) (2011). Microscopic esophagitis and Barrett’s esophagus: the histology report. Dig Liver Dis.

[8] Turato C, Pontisso P (2015). SERPINB3 (serpin peptidase inhibitor, clade B (ovalbumin), member 3). Atlas Genet Cytogenet Oncol Haematol.

[9] Schick C, Pemberton PA, Shi GP, Kamachi Y, Cataltepe S, Bartuski AJ, Gornstein ER, Brömme D, Chapman HA, Silverman GA (1998). Cross-class inhibition of the cysteine proteinases cathepsins K, L, and S by the serpin squamous cell carcinoma antigen 1: a kinetic analysis. Biochemistry.

[10] Suminami Y, Kishi F, Sekiguchi K, Kato H (1991). Squamous cell carcinoma antigen is a new member of the serine protease inhibitors. Biochem Biophys Res Commun.

[11] Pontisso P (2014). Role of SERPINB3 in hepatocellular carcinoma. Ann Hepatol.

[12] Vidalino L, Doria A, Quarta S, Zen M, Gatta A, Pontisso P (2009). SERPINB3, apoptosis and autoimmunity. Autoimmun Rev.

[13] Kato H, Torigoe T (1977). Radioimmunoassay for tumor antigen of human cervical squamous cell carcinoma. Cancer.

[14] Ciscato F, Sciacovelli M, Villano G, Turato C, Bernardi P, Rasola A, Pontisso P (2014). SERPINB3 protects from oxidative damage by chemotherapeutics through inhibition of mitochondrial respiratory complex I. Oncotarget.

[15] Collie-Duguid ES, Sweeney K, Stewart KN, Miller ID, Smyth E, Heys SD (2012). SerpinB3, a new prognostic tool in breast cancer patients treated with neoadjuvant chemotherapy. Breast Cancer Res Treat.

[16] Lim W, Kim HS, Jeong W, Ahn SE, Kim J, Kim YB, Kim MA, Kim MK, Chung HH, Song YS, Bazer FW, Han JY, Song G (2012). SERPINB3 in the chicken model of ovarian cancer: a prognostic factor for platinum resistance and survival in patients with epithelial ovarian cancer. PLoS One.

[17] Karamitopoulou E, Thies S, Zlobec I, Ott K, Feith M, Slotta-Huspenina J, Lordick F, Becker K, Langer R (2014). Assessment of tumor regression of esophageal adenocarcinomas after neoadjuvant chemotherapy: comparison of 2 commonly used scoring approaches. Am J Surg Pathol.

[18] di Pietro M, Lao-Sirieix P, Boyle S, Cassidy A, Castillo D, Saadi A, Eskeland R, Fitzgerald RC (2012). Evidence for a functional role of epigenetically regulated midcluster HOXB genes in the development of Barrett esophagus. Proc Natl Acad Sci USA.

[19] Stairs DB, Nakagawa H, Klein-Szanto A, Mitchell SD, Silberg DG, Tobias JW, Lynch JP, Rustgi AK (2008). Cdx1 and c-Myc foster the initiation of transdifferentiation of the normal esophageal squamous epithelium toward Barrett’s esophagus. PLoS One.

[20] Hao Y, Triadafilopoulos G, Sahbaie P, Young HS, Omary MB, Lowe AW (2006). Gene expression profiling reveals stromal genes expressed in common between Barrett’s esophagus and adenocarcinoma. Gastroenterology.

[21] Kim SM, Park YY, Park ES, Cho JY, Izzo JG, Zhang D, Kim SB, Lee JH, Bhutani MS, Swisher SG, Wu X, Coombes KR, Maru D (2010). Prognostic biomarkers for esophageal adenocarcinoma identified by analysis of tumor transcriptome. PLoS One.

[22] Kimchi ET, Posner MC, Park JO, Darga TE, Kocherginsky M, Karrison T, Hart J, Smith KD, Mezhir JJ, Weichselbaum RR, Khodarev NN (2005). Progression of Barrett’s metaplasia to adenocarcinoma is associated with the suppression of the transcriptional programs of epidermal differentiation. Cancer Res.

[23] Wang S, Zhan M, Yin J, Abraham JM, Mori Y, Sato F, Xu Y, Olaru A, Berki AT, Li H, Schulmann K, Kan T, Hamilton JP (2006). Transcriptional profiling suggests that Barrett’s metaplasia is an early intermediate stage in esophageal adenocarcinogenesis. Oncogene.

[24] Quarta S, Vidalino L, Turato C, Ruvoletto M, Calabrese F, Valente M, Cannito S, Fassina G, Parola M, Gatta A, Pontisso P (2010). SERPINB3 induces epithelial-mesenchymal transition. J Pathol.

[25] Gaur P, Hunt CR, Pandita TK (2016). Emerging therapeutic targets in esophageal adenocarcinoma. Oncotarget.

[26] Courrech Staal EF, Aleman BM, Boot H, van Velthuysen ML, van Tinteren H, van Sandick JW (2010). Systematic review of the benefits and risks of neoadjuvant chemoradiation for oesophageal cancer. Br J Surg.

[27] Tepper J, Krasna MJ, Niedzwiecki D, Hollis D, Reed CE, Goldberg R, Kiel K, Willett C, Sugarbaker D, Mayer R (2008). Phase III trial of trimodality therapy with cisplatin, fluorouracil, radiotherapy, and surgery compared with surgery alone for esophageal cancer: CALGB 9781. J Clin Oncol.

[28] van Hagen P, Hulshof MC, van Lanschot JJ, Steyerberg EW, van Berge Henegouwen MI, Wijnhoven BP, Richel DJ, Nieuwenhuijzen GA, Hospers GA, Bonenkamp JJ, Cuesta MA, Blaisse RJ, Busch OR, CROSS Group (2012). Preoperative chemoradiotherapy for esophageal or junctional cancer. N Engl J Med.

[29] Meredith KL, Weber JM, Turaga KK, Siegel EM, McLoughlin J, Hoffe S, Marcovalerio M, Shah N, Kelley S, Karl R (2010). Pathologic response after neoadjuvant therapy is the major determinant of survival in patients with esophageal cancer. Ann Surg Oncol.

[30] Cataltepe S, Gornstein ER, Schick C, Kamachi Y, Chatson K, Fries J, Silverman GA, Upton MP (2000). Co-expression of the squamous cell carcinoma antigens 1 and 2 in normal adult human tissues and squamous cell carcinomas. J Histochem Cytochem.

[31] Kato H (1996). Expression and function of squamous cell carcinoma antigen. Anticancer Res.

[32] Qi Y, Chiu JF, Wang L, Kwong DL, He QY (2005). Comparative proteomic analysis of esophageal squamous cell carcinoma. Proteomics.

[33] Yang YF, Li H, Xu XQ, Diao YT, Fang XQ, Wang Y, Zhao DL, Wu K, Li HQ (2008). An expression of squamous cell carcinoma antigen 2 in peripheral blood within the different stages of esophageal carcinogenesis. Dis Esophagus.

[34] Catanzaro JM, Sheshadri N, Pan JA, Sun Y, Shi C, Li J, Powers RS, Crawford HC, Zong WX (2014). Oncogenic Ras induces inflammatory cytokine production by upregulating the squamous cell carcinoma antigens SerpinB3/B4. Nat Commun.

[35] O’Riordan JM, Abdel-latif MM, Ravi N, McNamara D, Byrne PJ, McDonald GS, Keeling PW, Kelleher D, Reynolds JV (2005). Proinflammatory cytokine and nuclear factor kappa-B expression along the inflammation-metaplasia-dysplasia-adenocarcinoma sequence in the esophagus. Am J Gastroenterol.

[36] Tselepis C, Perry I, Dawson C, Hardy R, Darnton SJ, McConkey C, Stuart RC, Wright N, Harrison R, Jankowski JA (2002). Tumour necrosis factor-alpha in Barrett’s oesophagus: a potential novel mechanism of action. Oncogene.

[37] Turato C, Cannito S, Simonato D, Villano G, Morello E, Terrin L, Quarta S, Biasiolo A, Ruvoletto M, Martini A, Fasolato S, Zanus G, Cillo U (2015). SerpinB3 and Yap Interplay Increases Myc Oncogenic Activity. Sci Rep.

[38] Katagiri C, Iida T, Nakanishi J, Ozawa M, Aiba S, Hibino T (2010). Up-regulation of serpin SCCA1 is associated with epidermal barrier disruption. J Dermatol Sci.

[39] Pontisso P, Martini A, Turato C (2014). Liver pro-oncogenic potential of SERPINB3. Oncoscience.

[40] Ullman E, Pan JA, Zong WX (2011). Squamous cell carcinoma antigen 1 promotes caspase-8-mediated apoptosis in response to endoplasmic reticulum stress while inhibiting necrosis induced by lysosomal injury. Mol Cell Biol.

[41] Rugge M, Zaninotto G, Parente P, Zanatta L, Cavallin F, Germana B, Macri E, Galliani E, Iuzzolino P, Ferrara F, Marin R, Nisi E, Iaderosa G (2012). Barrett’s esophagus and adenocarcinoma risk: the experience of the North-Eastern Italian Registry (EBRA). Ann.Surg.

[42] Rhodes DR, Kalyana-Sundaram S, Mahavisno V, Varambally R, Yu J, Briggs BB, Barrette TR, Anstet MJ, Kincead-Beal C, Kulkarni P, Varambally S, Ghosh D, Chinnaiyan AM (2007). Oncomine 3.0: genes, pathways, and networks in a collection of 18,000 cancer gene expression profiles. Neoplasia.

